# Agile Development and Testing of a Gamified Human Milk Feeding Education Mobile App for Participants of the Special Supplemental Nutrition Program for Women, Infants, and Children: Co-Design Approach

**DOI:** 10.2196/80330

**Published:** 2026-04-29

**Authors:** Yarisbel Melo Herrera, Shannon Mitchell, Amy Malinowski, Catherine Wright, Marguerite Dibble, Patricia Cassi, Naina Qayyum, Erin Hennessy

**Affiliations:** 1 Gerald J. and Dorothy R. Friedman School of Nutrition Science and Policy Tufts University Boston, MA United States; 2 GameTheory Co Burlington, VT United States; 3 Special Supplemental Nutrition Program for Women, Infants, and Children (WIC) Vermont Department of Health Waterbury, VT United States

**Keywords:** agile development, app, breastfeeding, gamification, human milk feeding, mHealth, software testing, WIC

## Abstract

**Background:**

Human milk feeding and breastfeeding are the recommended standards for infant feeding. Nevertheless, breastfeeding rates in the United States remain below target levels, with disparities across racial, ethnic, and income groups. The Special Supplemental Nutrition Program for Women, Infants, and Children (WIC) plays a substantial role in reducing these disparities by providing lactation support to individuals with low income. With ongoing WIC modernization efforts, there is an opportunity to create and optimize technology solutions responsive to WIC participants’ and staff’s needs to increase access to the program and its services.

**Objective:**

This study aimed to describe the development and pilot testing of Daily Drop, a gamified, low-bandwidth mobile app to provide human milk feeding education and support for WIC participants.

**Methods:**

Guided by the 5-stage model for comprehensive research on telehealth, Daily Drop underwent 3 stages: concept development, service design, and preimplementation. Using a mixed methods approach, the project team sought feedback from WIC leadership and staff at the state and local levels, state IT staff, and WIC participants at each stage. Suggestions from stages 1 and 2 were incorporated into the testable app before field testing (stage 3). During field testing, participants and staff completed surveys across multiple time points and qualitative interviews to evaluate the app’s feasibility, usability, and acceptability. Quantitative data were analyzed using descriptive statistics, and qualitative data were thematically analyzed.

**Results:**

Key feedback from WIC participants and staff included providing flexibility for a variety of human milk feeding approaches (stage 1); and providing easily accessible educational information throughout gameplay, diversifying progress tracking to emphasize knowledge growth and expertise development, and including supportive or affirming messages for users (stage 2). During field testing (stage 3), >67% of WIC participants agreed with 7 out of 12 acceptability, satisfaction, and usability questions about the app, reiterated in interviews where they highlighted the simplicity of the app and how it increased their confidence to feed human milk. However, barriers to app use included a lack of reminders and repetitive information for parents with previous human milk feeding experience. Similarly, for WIC staff, mean scores for acceptability and feasibility were 3.8 (SD 1.0) and 4.4 (SD 0.6), respectively (max 5) at the early phase, but these scores declined over time. Staff recommendations included providing further, in-depth training to increase their familiarity with the app and reporting, and integrating the reports into WIC’s management information system.

**Conclusions:**

The development of Daily Drop followed an agile development, co-design approach with the involvement of relevant key partners at all stages of development. Overall, Daily Drop was deemed acceptable, usable, and engaging by WIC participants and staff. Future research could focus on testing its effectiveness in improving human milk feeding behaviors and cost-effectiveness.

## Introduction

### Overview

Breastfeeding and human milk feeding [1] are the recommended standards for infant feeding, given their short- and long-term health benefits for both the infant and birthing parent [2]. More specifically, the World Health Organization and the American Academy of Pediatrics recommend exclusive human milk feeding for the first 6 months of life, with continued human milk feeding alongside complementary solid foods until 2 years or beyond [2-4]. Nevertheless, rates of exclusive human milk feeding in the United States remain below Healthy People 2030 targets—by 15.2% at 6 months and 14.6% at 12 months [5-7]—and significant disparities exist across racial, ethnic, and income groups [5]. There is strong and consistent evidence of barriers impeding continued human milk feeding, including a lack of knowledge about human milk feeding, a lack of parental leave and unsupportive work environments, and a lack of access to health care or unsupportive health care practices, among others [8-10].

Therefore, one of the main goals of the Special Supplemental Nutrition Program for Women, Infants, and Children (WIC) is to provide lactation support, through access to lactation health professionals and peer counselors [11], to encourage human milk feeding initiation and extended duration among families with low income, and by extension, help reduce racial and ethnic disparities in these outcomes [12,13].

Nevertheless, as access to and use of technology and web-based and mobile apps has become more widespread in the United States [14], many parents have turned to these technologies to access information on human milk feeding [15,16]. Furthermore, the COVID-19 pandemic accelerated the shift toward online platforms and solutions within the WIC program, including remote nutrition education and lactation support appointments (eg, via phone or video call), remote benefit issuance, online nutrition education platforms, and online grocery ordering [17-19]. Since then, several reports have found that WIC participants accept and support routine technology use to better access WIC services [20,21]. Therefore, the US Department of Agriculture (USDA) Food and Nutrition Service launched WIC modernization efforts, which include initiatives to improve information management systems, create and optimize data-sharing solutions, and enhance websites and apps [22].

Recent systematic investigations of mobile health (mHealth) apps and interventions targeting human milk feeding behaviors among the general and low-income populations show that while apps are highly used and acceptable to users, their effectiveness on improving breastfeeding outcomes is highly variable [23-26]. This variability is likely due to significant heterogeneity in the app’s purpose (eg, provision of information vs tracking), active components (eg, videos, push notifications, and forum discussions), and target users (eg, birthing parents vs fathers), among other factors [23,26]. Furthermore, less is known about the extent to which end users were involved in the development process of these apps and the impact of this involvement on breastfeeding outcomes. Among studies evaluating user feedback, key components of apps include personalized education, peer support [25,26], encouragement, and audiovisual depictions of proper breastfeeding techniques [16]. Thus, incorporating user feedback during the mHealth app development is imperative to ensure maximum acceptability and uptake among intended users.

Agile development is a creative methodology that centers the perspective of a product’s audience through iterative testing and flexible development that adapts to new opportunities and challenges [27]. Key principles of agile development include (1) cross-disciplinary collaboration between audience members, subject matter experts, and creative professionals; (2) rapid, iterative development for early and frequent product testing; and (3) intentional reflection and decision-making through transparent testing, communication, and collaboration across key partner groups. Although agile development has widespread adoption across technology fields and has been shown to promote clarity, stability, and relevancy of products [28], it is still an underused process in many fields and programs, including WIC [29].

To our knowledge, no mHealth app focusing on human milk feeding behaviors has been developed specifically for WIC participants. Therefore, as part of the USDA/Tufts Telehealth Intervention Strategies for WIC (THIS-WIC) grant, the Vermont State WIC agency (VT WIC) collaborated with GameTheory Co to develop Daily Drop, a low-bandwidth mHealth app for WIC participants to use during pregnancy and through the early weeks postpartum for human milk feeding support. Daily Drop used a “serious game” design approach, which capitalizes on “traditional” game mechanics and technologies to promote learning and practicing skills, and by extension, evoke behavior change [30]. Consistent with an agile development approach, app content and functionality were informed by evidence-based WIC guidelines and refined iteratively through collaboration with subject matter experts, WIC leadership, and WIC participants. This manuscript reports on the agile development and pilot testing of Daily Drop.

### Conceptual Framework: 5-Stage Model for Comprehensive Research on Telehealth

**Figure 1:**
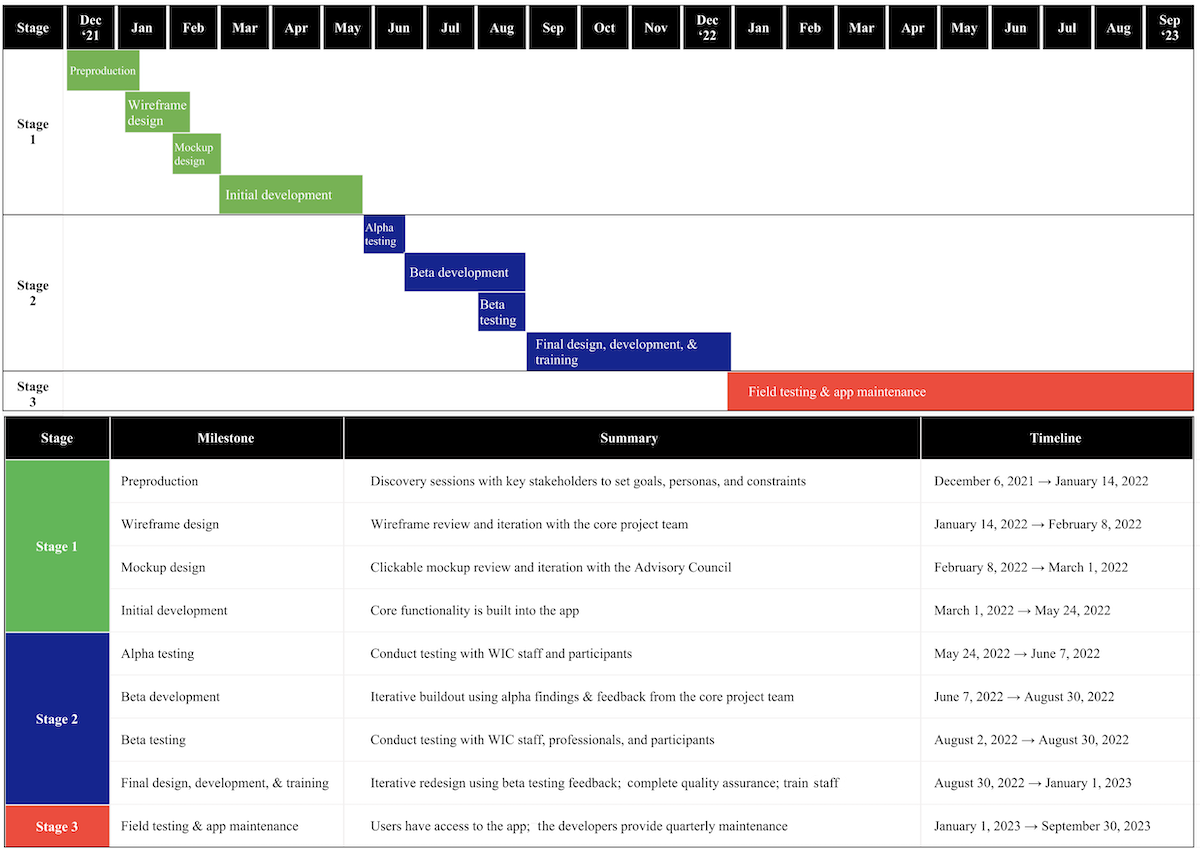
Timeline of the agile codevelopment process and testing of Daily Drop, a gamified human milk feeding education app for Special Supplemental Nutrition Program for Women, Infants, and Children (WIC) participants, in alignment with the 5-stage model for comprehensive research on telehealth.

The 5-stage model for comprehensive research on telehealth [31] informed the development and pilot testing of Daily Drop. The stages include (1) concept development, (2) service design, (3) preimplementation, (4) implementation, and (5) postimplementation. This manuscript reports the methodology and results of the first 3 stages (Figure 1 [31]), by stage. Throughout all stages, the core project team used respect-based design principles, which bridge elements of agile, user-centered, and empathetic co-design practices into a centralized theory for creating an mHealth solution that feels approachable, relevant, and most importantly, respectful of users’ identities and experiences.

### Stage 1: Preproduction, Design, and Initial Development

#### Methods

##### Preproduction

During this phase (December 2021 to January 2022), core project team members (software development team and VT WIC leadership) envisioned possibilities for the project, set goals and key performance indicators, and identified constraints, assumptions, and essential requirements for project success. Furthermore, to inform all aspects of the project, VT WIC convened an advisory council that included individuals representing the State of Vermont IT staff, VT WIC IT department, local agency WIC staff, and WIC participants. The core project team met with the advisory council to gather their insights on potential users, app benefits and risks, and to receive feedback on the alpha-, beta-, and field-testing plans, as well as evaluation-related questions.

**Figure 2:**
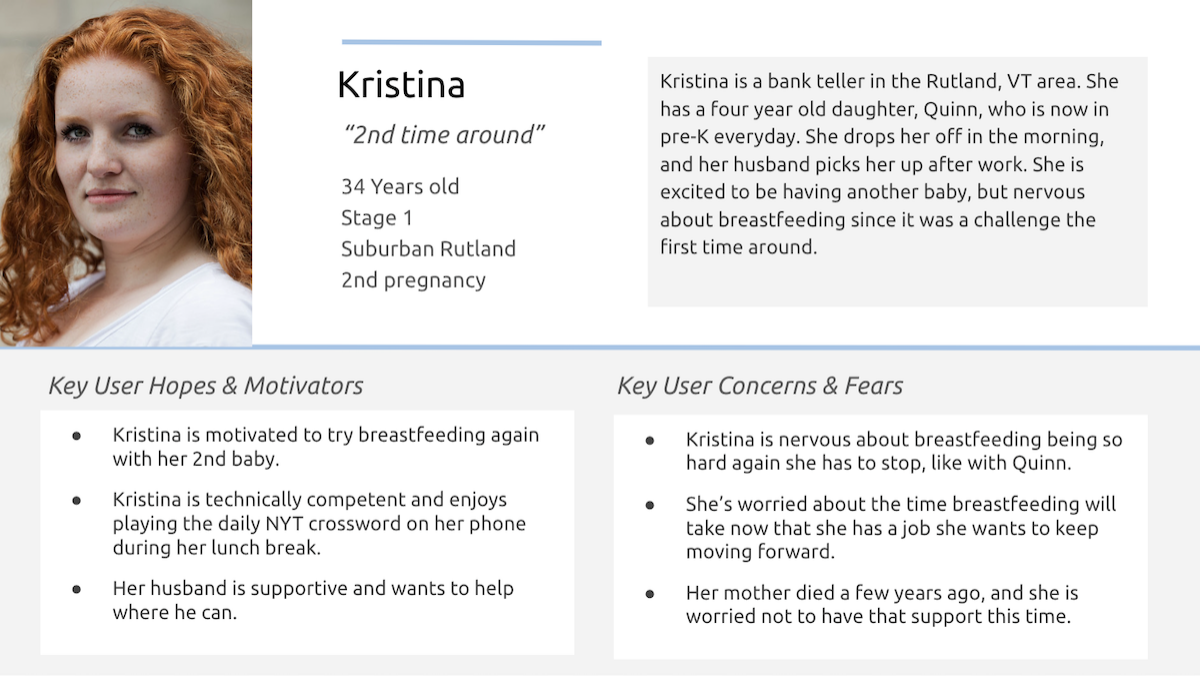
Example of a User Persona and Story used in Daily Drop’s preproduction (stage 1, December 2021 to January 2022). Image source: Flickr, published under Creative Commons Attribution 4.0 International License [36].

Using this input, the software development team developed “User Personas,” fictional profiles leveraged in user-centered design to describe potential users’ motivations, concerns, needs, and values [32]. User personas are widely used in health care and in the co-design of mHealth and eHealth technologies [32-34] to ensure that the proposed concept and planned functionality align with the needs of diverse potential users, rather than relying solely on the perspectives of those directly involved in the design process. For this project, User Personas were collaboratively designed to represent a wide range of perspectives, including technology access limitations, minimal baseline knowledge of the learning material, and socioeconomic characteristics and experiences not well represented within the core project team or the advisory council. Then, the software development team created accompanying “User Stories,” short feature descriptions written from a user perspective with information on the value each feature may provide to a specific User Persona (Figure 2) [35]. Together, the User Personas and User Stories provided a shared reference point for key partners in this stage and, in later stages, served as an evaluative framework to assess how the product design mapped against the range of qualities, experiences, sentiments, and perceptions of both potential end users and key partner groups in later stages.

Lastly, VT WIC leadership served as subject matter experts to identify key sources of evidence-based content for the app, which included USDA’s WIC Breastfeeding Curriculum and relevant resources from other state WIC agencies, USDA, Centers for Disease Control and Prevention, National Institutes of Health, La Leche League, and the Institute for the Advancement of Breastfeeding and Lactation Education.

##### Wireframe and Mockup Design

**Figure 3:**
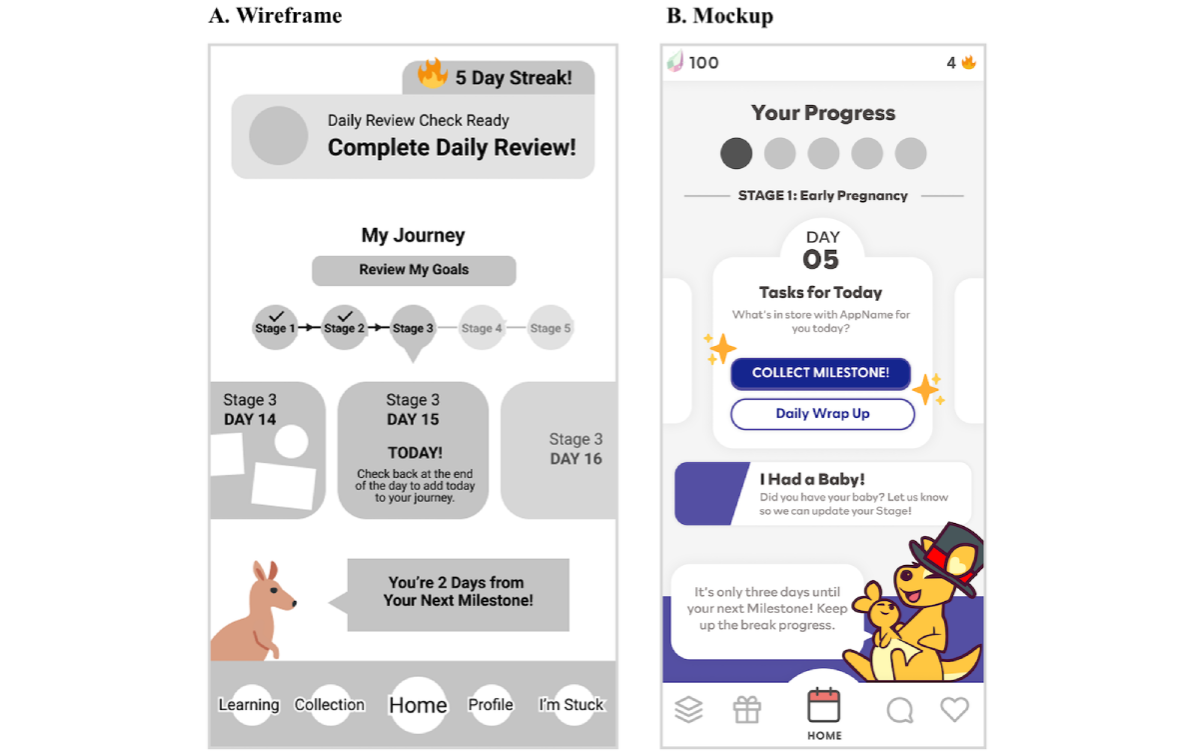
Wireframe (A) and clickable mockup (B) used in Daily Drop’s design (stage 1, January-February 2022).

This phase was conducted between January and March 2022. The software development team leveraged the findings from preproduction into a wireframing product screen by screen, without aesthetic or brand styling applied (Figure 3). The purpose of a wireframe is to define simple screens that can be quickly and easily changed based on review and feedback [37]. The software development team developed simple screen concepts based on the user stories and presented them to the core project team for review and feedback. The software development team also worked on stylistic development to establish a visual style and aesthetic approach for the product. Various style concepts were created and narrowed down based on the core project team’s feedback.

Together with the findings from the wireframe and style review sessions, the software development team refined and redeveloped the screens to create a clickable mockup to serve as a prototype (Figure 3). The clickable mockups were designed to showcase the desired product experience, intentionally excluding custom user input, final content, and any user data, thereby encouraging additional review. The software development team presented the clickable mockups to the advisory council for feedback early in the development process in order to identify areas for improvement.

##### Initial Development and Game Design

Following the wireframe and mockup design phase, the software development team worked to establish an early technical prototype between March and May 2022. In the manner of prototype, test, and evolve, the software development team created a horizontal slice, which focuses on building simple versions of the app features first. It excludes learning material and visuals to focus on the “must-have” features and systems. Once completed, the software development team included the key content selected by the subject matter experts to achieve an alpha level of completion. Software is considered alpha complete at the earliest point at which all core functionality and systems are ready for review by key partners.

#### Ethical Considerations

The Vermont Agency of Human Services Institutional Review Board (IRB) reviewed stage 1 activities and deemed them non–human subjects’ research. Still, the core project team reiterated to advisory council members that their contributions were voluntary and that they could stop their participation at any time. Advisory council members who were WIC participants received US $25 gift cards per meeting attended, whereas employees of the State of Vermont did not receive financial incentives in compliance with federal and/or state policies.

#### Results

##### Wireframe and Mockup Design

Key feedback at this phase included ensuring that games provided flexibility for a variety of human milk feeding approaches, allowing users to complete the introductory, setup questions for the app in an intuitive and easy way, increasing customization options, and leaning into the microlearning approach as an alternative to other digital information approaches. This feedback largely validated the initial wireframe design, while resulting in the design of a mascot with further customization options and game results that were neutral or open-ended rather than having strict right-or-wrong answers in all cases. The software development team applied the feedback from the advisory council to the clickable mockups and used the design as a blueprint as the project moved into the development phase. This early review served to refine and mitigate critical product risks at an early development stage, thus reducing overall project expenses.

##### Initial Development and Game Design

The User Personas established a preference for fast, pick-up-and-play experiences that could fit within busy schedules. Thus, the app was designed to operate through short microinteractions within a playlist, with rapid rewards for each interaction and the ability to advance game progress over relatively short durations. Minigames were selected for their recognizability and simplicity, relying on common interaction patterns such as drag-and-drop, matching, image comparison, and multiple-choice. These interaction styles reflect widely used user experience heuristics and were intended to reduce cognitive load while supporting a wide range of human milk feeding learning content.

Based on these design principles, 6 types of minigames were developed: matching games (eg, matching images and text), identifying differences in images, card sorting, and games to identify myths or facts about human milk feeding. When users opened the app, they accessed a “Daily Playlist” consisting of 5 minigames, enabling progress through short, self-contained sessions. User performance was tracked through points awarded for correct responses and deductions for incorrect answers.

At the start of the app experience, users completed the Breastfeeding Attrition Prediction Tool (BAPT) [38], a validated survey to assess readiness and supports around human milk feeding among pregnant individuals routinely used during lactation services by VT WIC [1,39]. The gamification pathway was personalized based on the user’s BAPT score domains, which relate to an individual’s knowledge of human milk feeding, support, and confidence. Each minigame was tagged to 1 of the 3 BAPT domains, and domain scores could range between –50 and 50 points depending on user responses. For the first 10 active days of gameplay, the “Daily Playlist” presented mostly minigames tagged to the user’s lowest domain score. After the first 10 days, minigames were randomly selected from a list of the 10 least recently shown minigames across all domains, and the 10 least recently shown minigames within the user’s lowest domain, supporting both reinforcement and content variety.

To reinforce app engagement, the app’s reward framework was informed by the Quantic Foundries Gaming Motivation Framework [40], leveraging “Completion” and “Achievement” motivation clusters. Completionist users are motivated to finish everything the game has to offer, for instance, complete every mission, find every collectible, and unlock all content. This motivation cluster has the broadest appeal across age and gender and aligns well with pick-up-and-play designs that unfold over multiple sessions. In Daily Drop, completionist engagement was supported through in-game rewards, including “Gems” earned for correct responses and redeemable for mascot costumes and accessories, randomized item unlocks, item rarity, playlist performance progress tracking and scoring, a daily emotion log, a knowledge library, and a minigame library. Additional commercially successful engagement mechanics were incorporated into the app, including streak tracking and a “Challenge Playlist” that allowed users to retry higher-difficulty content.

### Stage 2: Alpha and Beta Testing

#### Methods

##### Alpha Testing

Alpha testing is an assessment typically done in-house as a quality check to ensure accessibility, usability, accuracy of information, and alignment with technical, legal, and ethical standards [41]. However, to remain consistent with the agile development approach, the software development team invited potential users and key partners between May and June 2022 to respond to an early version of the project to identify potential access and usage barriers that could limit product impact [42]. Alpha testing procedures included conducting 4 focus groups via video call with 2 groups: WIC participants (n=6) and WIC staff, including full-time nutritionists (n=2) and breastfeeding peer counselors (n=1). WIC staff in 5 local offices recruited WIC participants who met the inclusion criteria (aged 18 years or older and pregnant) during routine appointments. If WIC participants were interested in taking part in alpha testing, they provided their contact information, which was shared with the project team. A project team member then reached out to formally invite them to participate. The project team also emailed all WIC staff and breastfeeding peer counselors to invite them to participate in the focus groups.

Each focus group had 1-2 moderators and an observer. Focus groups began with an overview of the project, a demo of the early prototype, and examples of the planned look and feel. Then, focus group participants were asked questions related to (1) experiences with human milk feeding, (2) current technology use and barriers to technology use, (3) preferences on the art style, (4) the alignment of the prototype with project goals and staff and user needs, and (5) overall suggestions for improvements. Finally, focus group participants completed a survey regarding app usability and motivations for future use.

##### Beta Development

Following alpha testing, the software development team incorporated feedback through weekly development, testing, and review with the core project team between June and August 2022. Changes and updates were checked against the available project scope before being applied. If any changes were deemed out of scope, design work was completed to identify alternative mechanisms to address the underlying feedback. The product gradually achieved greater stability through this process, and its functionality was expanded and refined until it reached a nearly final state. Quality assurance testing was also conducted toward the end of the beta development phase to identify any high-priority issues that need resolution before beta testing [43-45]. Examples of procedures included testing the app with older phones or unstable internet connection and ensuring appropriate functionality for all app features.

##### Beta Testing

Beta testing aims to validate the product in real-world conditions before release, evaluating user experiential learning and interactivity to ensure alignment with the project’s goals [41]. In August 2022, WIC participants (n=12), staff (n=5), and professionals (ie, WIC experts outside of VT WIC, n=5) served as beta testers. Some WIC participants who were alpha testers were invited to participate as beta testers, in addition to new testers. WIC staff recruited new WIC participant testers during routine appointments at 5 local offices, provided they met the inclusion criteria (aged 18 years or older and currently or recently pregnant). If WIC participants were interested in taking part in beta testing, they provided their contact information, which was shared with the project team. A project team member then reached out to formally invite them to participate. WIC participants were involved in beta testing over a 4-week period, which included 2 meetings with asynchronous testing sessions in between.

The first meeting of beta testing was an onboarding session for groups of no more than 4-6 WIC participants. During this meeting, the software development team walked WIC participants through downloading the game onto their smartphones, demonstrated how to use the app, and discussed the testing purpose in this phase. WIC participants were given access to the game and asked to use it for 4 weeks. While testing, data on their app usage was collected and linked to their WIC ID. Collected metadata included time spent in the app, game engagement streaks (ie, consecutive days using the app), and scores in each BAPT domain (knowledge, support, and confidence). At the close of the testing window, the VT WIC team conducted qualitative interviews with each WIC participant via video call. Interview questions asked about testers’ barriers and facilitators to using the product, motivators for future use, and overall experience. After the interview, WIC participants also completed a survey (developed by the core project team) regarding the acceptability and ease of use of the app and changes in perceptions regarding their human milk feeding experience, informed by key feedback from alpha testing.

WIC staff and professionals were invited to participate in beta testing via email. However, the instructions for testing were more flexible for WIC staff and professionals than for participants, encouraging them to explore the app to become familiar with its features and educational framework. Then, WIC staff and professionals who tested the app were invited to complete the same survey as WIC participants. WIC staff did not receive financial incentives in compliance with federal and/or state policies.

##### Final Design, Development, and Staff Training

**Figure 4:**
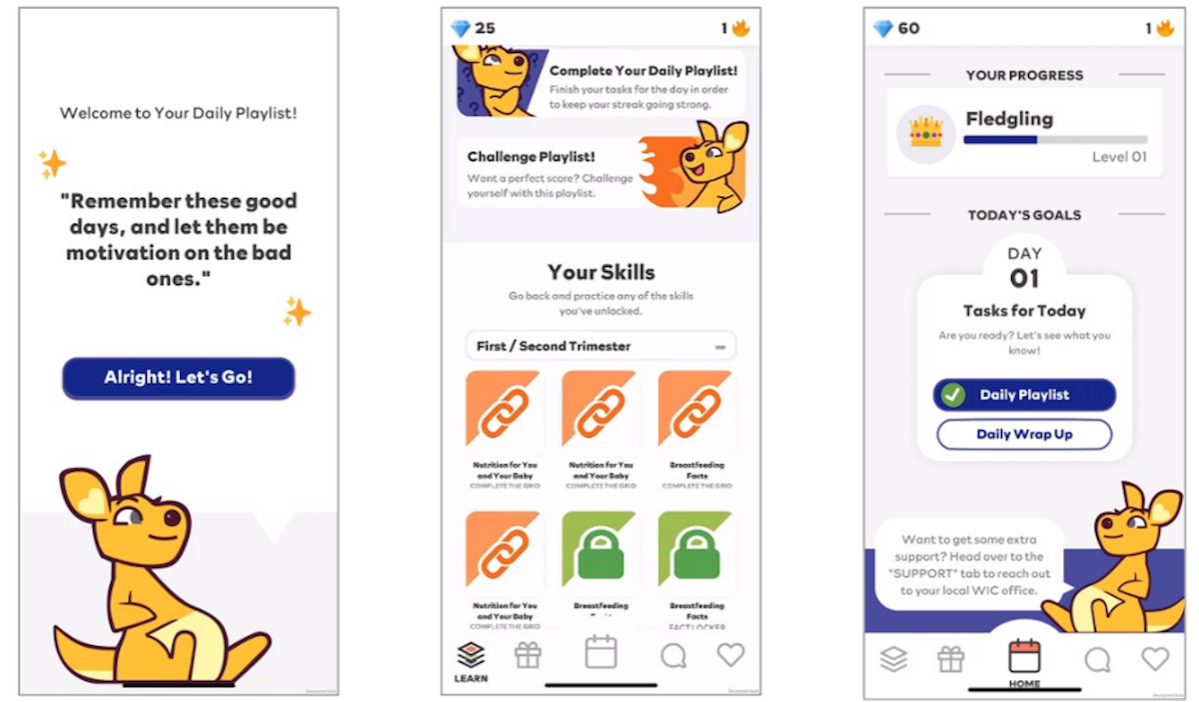
Screenshots of Daily Drop’s final design (stage 2, September-December 2022). From left to right: (A) initial screen with motivational message and the app’s mascot, (B) overview of the “Learn” tab, allowing access to Daily and Challenge Playlists and skills library, (C) overview of the “Home” tab, displaying daily tasks and progress, with a message on how to connect with the local WIC office.

The core project team collaborated in a final development phase for approximately 4 months (August 2022 to January 2023) to design upgrades and changes to the app based on the findings of beta testing. These designs were built into the app, and the software development team completed additional iterative rounds to ensure the user-identified issues were addressed. Lastly, the team completed a rigorous quality assurance phase during which the tool was tested on various devices and conditions to ensure the app was stable and ready for the pilot trial (Figure 4).

During this phase, the core project team solidified the data transfer and storage processes, which included establishing the file structure, creating the data dictionary, and developing a data visualization dashboard. The dashboard was vetted with the advisory council and revised based on their feedback. This dashboard was used to track key components of users’ app experience, such as time in app, time per session, and changes in learning scores during the field-testing stage (stage 3).

Lastly, the core project team hosted a 2-part training for WIC staff in preparation for the field-testing stage. Part 1 included introducing the app, its purpose and features, a preview of the clinical report tool, and instructions on how to download and use the test app. Part 2 included providing details on the pilot trial and their role in it, how to promote the app and document its use in WIC’s management information system, and practice using the clinical report tool in breakout rooms.

##### Quantitative Data Analysis

Analyses were conducted in SAS (version 9.8; SAS Institute). Descriptive statistics were performed to summarize responses to alpha- and beta-testing survey items (via means and SDs) among WIC participants, staff, and professionals.

##### Qualitative Data Analysis

A thematic analysis [46] of alpha testing focus groups was conducted using a deductive approach. Two members of the software development team developed a codebook and summary template in alignment with focus group questions (behavioral motivations for app engagement, validation of design and mechanical choices, content suggestions, and functionality requests), and summarized the content of focus groups transcripts into the template. To ensure trustworthiness and credibility of findings [47], and alignment with the project goals, themes, subthemes, and exemplar quotes were member-checked with the advisory council and members of the core project team and discussed until consensus was reached.

#### Ethical Considerations

The Vermont Agency of Human Services IRB reviewed stage 2 activities and deemed them non–human subjects’ research. Still, the core project team reiterated to testers that participation was voluntary, and that they could stop their participation at any point. WIC participants who attended alpha testing focus groups received a US $25 gift card, and those who completed all portions of beta testing received US $100 gift cards in compensation for their time. WIC staff did not receive financial incentives for their participation in stage 2 activities in compliance with federal and/or state policies.

#### Results

##### Alpha Testing

Survey results indicated that alpha testers perceived the app as very useful for pregnant parents both in rural Vermont and overall, and indicated that “building skills and knowledge” and “having a quick way to get answers to questions or help with challenges” were the most prominent factors to make them interested in using the app (data not shown). Key suggestions from focus groups included (1) allowing app users to complete the BAPT questionnaire within the first couple of days using the app vs completing it during sign-up due to its length, (2) providing educational information after incorrectly answering questions, and (3) adding affirmations throughout gameplay to encourage users and keep the app feeling supportive.

##### Beta Testing

Survey results indicated that all WIC participants and staff considered their app experience as positive (mean scores 5.0-6.0 out of 7), highlighting the app’s potential to improve the breastfeeding experience (mean scores 5.4-6.0 out of 7; Table 1). However, the mean score of 4.0 for the statement “Was the application motivating for you to keep playing over time?” was indicative of neutral motivation for WIC participants. This finding was supported during qualitative interviews, during which WIC participants indicated wanting the app to provide more answer clarity and content diversity. This feedback led to the following improvements: (1) diversification of progress tracking or reward mechanics, particularly emphasizing knowledge growth and expertise development, (2) adjustments to the variety and length of the daily content, (3) improvements to navigation and user experience, and (4) updates to improve user experience on a broader range of mobile devices.

**Table 1:** Post–beta testing of Daily Drop survey results among WIC^a^ participants, staff, and professionals (stage 2, August 2022).

Statement	WIC^a^ participants (n=12), mean (SD)	WIC staff (n=5), mean (SD)	WIC professionals (n=5), mean (SD)
Overall, how would you rate your experience with the application?^b^	5.3 (0.9)	6.0 (0.6)	5.0 (0.7)
How easy was it to find the information you wanted in the application?^c^	4.8 (1.9)	5.4 (1.0)	5.4 (1.3)
Was the application motivating for you to keep playing over time? (WIC participants)^d^	4.0 (1.3)	5.2 (0.4)	4.8 (1.1)
How motivating did you perceive the application would be for WIC participants? (WIC staff and professionals)^d^	4.0 (1.3)	5.2 (0.4)	4.8 (1.1)
How do you feel playing the application when you were pregnant/breastfeeding would have influenced your breastfeeding experience? (WIC participants)^e^	5.7 (1.2)	6.0 (0.9)	5.4 (0.55)
How do you feel a WIC user’s breastfeeding experience would have improved or worsened because of the application? (WIC staff and professionals)^e^	5.7 (1.2)	6.0 (0.9)	5.4 (0.55)
Was the amount of breastfeeding information presented in the daily playlist an appropriate amount for your learning?^f^	5.2 (0.9)	N/A^g^	N/A
How do you feel your ability to provide breastfeeding education/counseling would be improved or worsened because of the application?^e^	N/A	5.6 (0.8)	5.4 (0.5)

^a^WIC: Special Supplemental Nutrition Program for Women, Infants, and Children.

^b^7-point Likert scale where 1=very negative and 7=very positive.

^c^7-point Likert scale where 1=very hard and 7=very easy.

^d^7-point Likert scale where 1=very demotivating and 7=very motivating.

^e^7-point Likert scale where 1=greatly worsened and 7=greatly improved.

^f^7-point Likert scale where 1=not enough information and 7=too much information.

^g^N/A: not applicable.

### Stage 3: Field Testing (Pilot Trial)

#### Methods

##### Overview

The project team pilot tested the (1) acceptability, feasibility, and usability of Daily Drop among WIC participants and (2) the acceptability and usability of Daily Drop to support lactation services among WIC staff. Between January 2023 and September 2023, 5 WIC local agencies participated in the pilot. WIC participants were recruited using multiple methods. First, WIC staff recruited WIC participants during their routine appointments, provided they met the inclusion criteria (aged 18 years or older, currently or recently pregnant, and had access to a smartphone or tablet, n=339). If WIC participants were interested in taking part in field testing, they provided their contact information, which was shared with the project team. A project team member then reached out to formally invite them to participate, provided them with an electronic study information sheet, and obtained their consent. Second, WIC staff also posted physical flyers at the 5 local WIC offices and sent emails and SMS text message blasts inviting potentially eligible WIC participants. Interested WIC participants could contact the project team to receive an electronic study information sheet and consent to participate.

Upon consenting, WIC participants were invited to download the app on their mobile device and asked to provide information to guide them through gameplay. This information included WIC ID, local agency, preferred term (breastfeeding or chestfeeding), pregnancy status, anticipated due date, and baby’s birth date (if they indicated they recently gave birth). After the initial setup, users were asked to complete the BAPT questionnaire. Additionally, WIC staff responsible for delivering lactation support at participating agencies were invited to participate in the evaluation.

##### WIC Participants

WIC participants were asked to complete up to 3 surveys during the evaluation, including a baseline survey (n=74), a 1-month user survey (n=49; those who played Daily Drop at least once) or a 1-month nonuser survey (n=25), and a 3-month postpartum user survey (n=42). Electronic surveys with embedded consent language were programmed using Alchemer (Alchemer), and links were sent by VT WIC staff via email or SMS text message. Up to 2 reminders were sent for the baseline and 1-month surveys. One reminder was sent for the 3-month postpartum survey following the initial text/email. This manuscript presents results for questions related to acceptability, satisfaction, and usability among app users in the 1-month survey, which were adapted from valid and reliable tools [48-51]. Adaptations were necessary in this context, given that no existing tools captured all the aspects of WIC human milk feeding education, support, and lactation staff-participant interactions.

Moreover, a subset (n=26) of WIC participants who used Daily Drop were invited to an interview at the end of the evaluation period to provide additional insights about their app use. This subset was selected using random stratified sampling based on their app use (n=13 per stratum; stratum 1: low use [fewer than 10 sessions]; stratum 2: moderate to high use [10 or more sessions]). The semistructured interview guide was tested with members of the advisory council and refined based on their feedback. Interviews were scheduled for 30 minutes and digitally recorded and transcribed. Interviews were conducted until saturation was reached (ie, no new emergent themes) [52].

##### WIC Staff

Eligible WIC staff (n=24) were invited to participate in the evaluation via email with an embedded survey link. Staff surveys with embedded consent information were distributed electronically via Qualtrics (Qualtrics) 3 times during the evaluation: once in the early phase after project implementation (January-February 2023; n=16), once at the midphase (July 2023; n=13), and once in the late phase after the pilot concluded (October 2023; n=8). Up to 2 reminders were sent via email to eligible staff who did not complete a survey, 1 week and 2 weeks after the initial email invitation. Similar to WIC participant surveys, staff survey items were adapted from valid and reliable tools [48,53-55] to ensure that all the aspects of WIC human milk feeding education, support, and lactation staff-participant interactions were captured in responses.

Additionally, WIC staff and local agency directors at participating agencies were invited to participate in interviews. A semistructured interview guide, informed by the Reach, Effectiveness, Adoption, Implementation, and Maintenance [56] and the Consolidated Framework for Implementation Research (CFIR) [57] implementation frameworks to assess key implementation aspects (eg, relative advantage, compatibility, complexity, and trialability), was used to guide interviews. Interviews were conducted via video call in the early (March 2023, n=6), mid (August-September 2023, n=6), and late phases (October 2023, n=2) of the implementation period. The interviews were scheduled for 1 hour and were digitally recorded and transcribed. Given that there was a small number of eligible staff, the number of interviews at each phase was dictated by the number of staff willing to participate rather than saturation.

##### Quantitative Data Analysis

Analyses were conducted in Stata (version 18; StataCorp LLC). Descriptive statistics were performed to summarize acceptability, satisfaction, and feasibility (via frequencies and percentages, and means and SDs) among WIC participants and staff.

##### Qualitative Data Analysis

A thematic analysis [46] of WIC participants and staff interviews was performed using a deductive approach, with a preliminary codebook developed using CFIR [57] and the Evaluation Framework for Telemedicine [58] prior to analysis. A total of 10 trained qualitative researchers coded interview transcripts, with each transcript being independently coded by 2 researchers using NVivo (version 13; Lumivero). Coders met regularly to discuss coding disagreements, reach consensus, and update the codebook, if needed.

#### Ethical Considerations

For research activities in stage 3, The Vermont Agency of Human Services IRB reviewed and approved the protocol related to the collection of WIC participant data, including surveys, metadata from Daily Drop, and WIC’s management information system data (#308). The Tufts University IRB reviewed and approved the protocols related to WIC staff data, including surveys and qualitative interviews (#00003228). All individuals who took part in these research activities provided written informed consent, and all procedures were performed in accordance with the ethical standards of the approving IRBs and the 2013 Declaration of Helsinki.

WIC participants who used the app received a US $50 gift card for completing the baseline survey, a US $50 gift card for completing the 1-month survey, and a US $50 gift card for completing the qualitative interviews. WIC participants who did not use the app and completed the nonuser survey received a US $10 gift card. Incentives were not offered for the 3-month postpartum survey. WIC staff did not receive incentives for completing surveys or interviews, in compliance with federal and/or state policies.

#### Results

##### WIC Participants

The results of the 1-month user survey indicated that app users enjoyed using it (35/49, 71.4%), appreciated its simplicity (46/49, 93.9%), and improved their breastfeeding learning experience (39/49, 79.6%; Table 2). Users also expressed satisfaction with the app, with 71.4% (35/49) indicating playing Daily Drop was a good use of their time. About 89% (44/49) suggested WIC offices should use Daily Drop. About two-thirds (33/49, 67.4%) of respondents stated that Daily Drop allowed them to learn about breastfeeding more quickly and easily. Qualitative interviews largely supported these findings, with users discussing that it was “fun,” “helpful,” and “trustworthy.” Users mentioned that the app increased their self-efficacy: “It gave me more confidence in my ability because I had no idea how to breastfeed or if I was doing it right or even to know like if my baby was full. It gave a lot of knowledge that I didn’t have before.”

However, some users noted that the app might be less beneficial for individuals with previous human milk feeding experience, as the information could feel repetitive and may not provide as much value compared to first time parents: “I don’t think I would share it with somebody that’s had seven kids and breastfed all of them, but somebody that’s new to breastfeeding or new to just motherhood. I would share it because, like I said, there’s a lot of information on there that you can learn from it.” Lastly, the absence of push notification features and the inability to play more than one playlist per day were highlighted as barriers to using the app: “My only issue was remembering to do it because it didn’t have any reminders, or if it did, I didn’t have them on.”

**Table 2:** Acceptability and satisfaction with Daily Drop among WIC^a^ participants who completed the 1-month user survey (n=49; stage 3, January-September 2023).

Construct and statement			Strongly agree or agree, n (%)		Neither agree or disagree, n (%)		Strongly disagree or disagree, n (%)
**Acceptability**							
	I had more fun learning about breastfeeding because of using Daily Drop.	35 (71.4)		11 (22.4)		3 (6.1)	
	Using Daily Drop made learning about breastfeeding a better experience than it would have been otherwise.	39 (79.6)		8 (16.3)		2 (4)	
	I would like to play the Daily Drop more.	29 (59.2)		13 (26.5)		7 (14.3)	
	I learned about breastfeeding more quickly and easily because of using Daily Drop.	33 (67.4)		14 (28.6)		2 (4)	
	Playing Daily Drop made me want to use more WIC breastfeeding support resources.	23 (46.9)		18 (36.7)		8 (16.3)	
	Using Daily Drop made what I was learning from my IBCLC^b^ or peer counselor feel more relevant to me.	16 (32.7)		30 (61.2)		3 (6.1)	
	My WIC staff person provided helpful feedback based on my Daily Drop scores.	12 (24.5)		20 (40.8)		17 (34.7)	
**Satisfaction**							
	I am glad I played Daily Drop	38 (77.5)		9 (18.4)		2 (4)	
	Using the Daily Drop was a good use of my time.	35 (71.4)		11 (22.4)		3 (6.1)	
	I would recommend Daily Drop to other WIC participants.	41 (83.7)		6 (12.2)		2 (4)	
	I think other WIC offices should offer Daily Drop.	44 (89.8)		4 (8.2)		1 (2)	
**Ease of Use**							
	Daily Drop was simple for me to use.	46 (93.9)		2 (4.1)		1 (2)	

^a^WIC: Special Supplemental Nutrition Program for Women, Infants, and Children.

^b^IBCLC: International Board–Certified Lactation Consultant.

##### WIC Staff

WIC staff agreed that Daily Drop was an acceptable way to provide WIC services (Table 3). The average agreement score for the usefulness of the app declined over time, but the difference was not significant. Mean interest scores in using Daily Drop feedback for breastfeeding support were neutral in the midphase and low in the late phase. However, feasibility measures declined steadily over time. Early phase scores for learning gameplay feedback on breastfeeding education approached agreement on ease of use, while late-phase scores indicated disagreement. Average scores for understanding reports and experience with Daily Drop reports showed low feasibility for providing client feedback in the clinic.

These findings were further supported during qualitative interviews, where WIC staff mentioned that the app would benefit WIC participants due to its interactive features, which help deliver lactation information in an engaging manner. Staff also appreciated the self-paced nature of the app, which eased the burden of information overload often experienced during lactation support appointments:

We’re really excited about having another way to present breastfeeding information to parents...it’s user-friendly, and it’s tailoring to what families are, what information they’re looking for.

Early phase

Furthermore, staff indicated that BAPT scores and gameplay reports allowed them to provide even more tailored discussions and conversations based on the participants’ needs:

We use the (app) data mostly at the mid-certification visit for pregnant participants... We also use it to check our BAPT survey, which is done on the Daily Drop app also separately. And mostly it’s used as a tool to then follow up about topics that are either of peak interest or topics that don’t seem to be as much on a family’s radar.

Midphase

Nevertheless, during mid- and late-phase interviews, staff noted that more comprehensive training on using the app and integrating gameplay in the lactation appointments would have made it more likely for them to use it. Staff’s concerns were less about the app’s design and more about its implementation and their readiness to incorporate it into their routine WIC appointments:

And I do think that if we continue to use it, that at least I would get the hang of both reviewing it, reviewing those reports ahead of the appointments and incorporating it into the actual appointment itself pretty effectively.

Late phase

**Table 3:** Feasibility and acceptability of Daily Drop among WIC^a^ staff who completed the early- (March 2023), mid- (August-September 2023), and late-phase (October 2023) surveys (stage 3).

Construct and statement		Early phase (n=14), mean (SD)^b^	Midphase (n=6), mean (SD)^b^	Late phase (n=3), mean (SD)^b^
**Acceptability**				
	Daily Drop is an acceptable way to provide WIC breastfeeding education.	4.4 (0.6)	3.7 (0.5)	3.3 (0.6)
	Daily Drop reports are useful for me as WIC staff.	4.3 (0.7)	3.0 (1.3)	2.7 (1.5)
	I would like to continue using Daily Drop gameplay feedback in my breastfeeding support sessions.	N/A^b^	3.0 (1.1)	2.0 (1.0)
**Feasibility**				
	Learning to use gameplay feedback for breastfeeding education and support from Daily Drop was easy for me.	3.8 (1.0)	3.0 (1.1)	2.0 (1.7)
	I find the Daily Drop reports easy to understand.	3.9 (0.9)	2.8 (1.3)	3.0 (1.0)
	I have a lot of experience giving Daily Drop gameplay feedback in my clinic.	N/A	1.5 (0.5)	1.3 (0.6)
	I am good at giving Daily Drop gameplay feedback in my clinic appointments.	N/A	2.5 (1.4)	1.7 (1.2)

^a^WIC: Special Supplemental Nutrition Program for Women, Infants, and Children.

^b^Mean and SD are from 5-point Likert scales, where 1=strongly disagree and 5=strongly agree.

^b^N/A: not applicable.

## Discussion

### Overview

This manuscript describes the development and testing of a gamified, human milk feeding education app designed for pregnant and postpartum WIC participants. Consistent with the agile development approach [27], the core project team integrated mixed methods feedback from key partner groups, including WIC participants and staff, at all stages of app development (concept development, service design, and preimplementation [31]). Active user involvement is critical at these stages, as it can increase the perceived value of the mHealth product and future successful uptake [59]. This iterative, cyclical process can help ensure high-quality mHealth software is usable and acceptable for end users, increasing its potential to improve health-related behaviors and outcomes [60,61]. Furthermore, using a “serious game” design [30] enabled the transformation of extensive content into easily digestible, minigame formats—an innovative approach within the WIC context, compared to traditional methods of delivering lactation education and support.

Key feedback from WIC participants and staff included providing easily accessible educational information throughout gameplay, diversifying progress tracking to emphasize knowledge growth and expertise development, and including supportive or affirming messages to users, among others. This feedback was incorporated before the field-testing stage of development, and preliminary results from mixed methods evaluations with WIC participants and staff suggested that the app was acceptable, user-friendly, and engaging, and that the lactation information provided was easier to understand compared to more traditional education materials.

These findings align with previous research evaluating implementation outcomes (eg, acceptability, feasibility, and engagement) of mHealth apps targeting human milk feeding behaviors among the general and low-income populations. Several systematic reviews have shown that such apps are highly acceptable and have moderate to high levels of engagement [23-26]. Nevertheless, apps had varying levels of effectiveness in improving human milk feeding behaviors, often attributed to the heterogeneity of the apps’ engagement, whether the app focused on the provision of information vs interactive support (eg, from lactation consultants or peers) vs monitoring or tracking behaviors, and participant characteristics (eg, otherwise healthy pregnant individuals vs those with pregnancy complications). While this manuscript does not describe behavioral outcomes, it is worth emphasizing that apps may not be as effective in evoking behavior change in the absence of multipronged strategies to reduce the structural barriers to human milk feeding [10,62], particularly those faced by parents with low income. Given WIC’s eligibility requirement of medical or nutrition-related risks, participants may face additional or more severe human milk feeding challenges compared to income-eligible populations. Therefore, mHealth strategies seamlessly integrated into WIC services, paired with already established initiatives (lactation professionals, classes and support groups, peer counselors, among others [63]), have the potential to achieve higher reach and scalability among those who may need it most, and increase the program’s impact on reducing income, racial, and ethnic disparities in human milk feeding outcomes.

A recent systematic review showed that WIC participants consider mHealth diet interventions (eg, apps) highly acceptable and feasible, although no app focused solely on human milk feeding behaviors [64]. Furthermore, in various states, apps focusing on nutrition or lactation education can also be used to fulfill nutrition education requirements instead of attending in-person classes, further reducing transportation and scheduling-related barriers to WIC participation. In sum, our findings contribute to the growing evidence supporting the increased use of technologies within the WIC context to improve access to the program and its services and potentially improve health-related outcomes, ensuring WIC’s continued success in promoting the health and well-being of its participants.

### Limitations

The findings of this research must be interpreted in the context of the following limitations. First, the core project team used a convenience sampling approach, and sample sizes across all development and testing stages were fairly small (n<75), especially for WIC staff (n<14); thus, results may not be as generalizable to the larger VT WIC participant population or lactation staff. Nevertheless, this was proof-of-concept research, and preliminary results demonstrated the acceptability and usability of the app among WIC participants and staff. Therefore, a necessary next step for our research is to better understand how effective the app is at improving human milk feeding behaviors compared to usual care (eg, electronic or printed handouts) with a larger, more diverse sample of WIC participants.

Second, while survey items were adapted to the WIC context from valid and reliable tools and reviewed by subject matter experts, they were not validated with the target population, further limiting their generalizability. Additionally, the use of multiple assessment tools with varying Likert scale formats may introduce complexity in cross-measure comparisons. Thus, as mobile apps become more widely integrated into WIC routine care as a result of ongoing modernization efforts [22], future research should focus on developing and validating tools to standardize implementation outcomes in this context.

Lastly, the app was developed only in English. While 94% of VT WIC households list English as their preferred spoken language [50], future work is needed to ensure appropriate cultural and linguistic adaptations in the app to ensure fair access for all WIC participants.

### Lessons Learned

Several areas for improvement and lessons learned emerged from our process. From a logistics perspective, for software-related projects specifically for federal nutrition assistance programs, it is critical to ensure sufficient time is planned to procure and establish a contract with a vendor with expertise in leading a user-centered design project, as technical procurements within federally funded agencies involve additional levels of internal review to ensure security standards are met. Furthermore, developing and maintaining strong relationships with internal IT staff is crucial for implementing the necessary components within the government environment. These include facilitating the technical procurement review process, meeting government security standards, and establishing data flow and storage of game use data while preserving user privacy. Lastly, having a dedicated project management staff to coordinate with the vendor and internal government partners, assist with recruitment, and coordinate testers in each stage was key to our success.

From the WIC staff’s perspective, our results suggest that comprehensive training is crucial for equipping staff to demo the app to participants, access and interpret game reports, and use them during lactation support appointments, all while ensuring that these tasks do not substantially increase staff burden, which is critical for staff buy-in. Moreover, from the WIC participants’ perspective, engagement data suggested that users interacted with the app for a particular duration and reported experiencing benefits. Thus, explicitly communicating a specific time frame for app use (eg, how much, how often, and for how long) can help users feel motivated and perceive more benefits, potentially increasing the app’s impact on human milk feeding behaviors.

### Implications for Future Research and Practice

With the ongoing WIC modernization efforts [22], this research can serve as a guide for other state and local WIC agencies wanting to incorporate technology solutions into their routine practices. The co-design, user-centered approach involving WIC leadership, staff, participants, and software developers resulted in an acceptable and user-friendly app. It is worth noting that another key advantage of agile development and end-user feedback during the early stages relates to cost savings, particularly for government agencies, like WIC. “Traditional” software development usually involves a “waterfall” or “top-down” approach, during which end-user feedback is not incorporated until the last phase—when the software is operating in a live environment [28]. Contrarily, by gathering end-user feedback early and throughout all stages of development, and providing interactive materials (eg, early prototypes of the app) instead of “hypothetical” questions (eg, “what would you like to see in the app”?), software developers can ensure that the final product is closely aligned with end-user requirements, thus increasing the likelihood of greater acceptability and engagement and decreasing the need for costly updates once the product is “live.” Nevertheless, further research evaluating the cost-effectiveness of these technologies is warranted.

### Conclusions

Daily Drop is a gamified mobile app that provides human milk feeding education and support specifically for WIC participants. Using an agile development and co-design approach involving key partner groups at all stages of development, Daily Drop was deemed acceptable, usable, and engaging by end users. Future research could focus on statewide implementation, testing in a randomized controlled trial, and evaluating the cost-effectiveness of the app. The widespread implementation of mHealth solutions in the WIC program is a vital step forward into the increasingly digital health landscape, to support WIC’s mission of improving the health and well-being of families with low income.

## Abbreviations

BAPT: Breastfeeding Attrition Prediction Tool
CFIR: Consolidated Framework for Implementation Research
HIPAA: Health Insurance Portability and Accountability Act
IRB: institutional review board
mHealth: mobile health
THIS-WIC: USDA/Tufts Telehealth Intervention Strategies for WIC
USDA: US Department of Agriculture
VT WIC: Vermont State WIC agency
WIC: Special Supplemental Nutrition Program for Women, Infants, and Children

## Data Availability

The datasets generated and analyzed during this study are not publicly available. Data were collected and shared between the Trustees of Tufts College and the State of Vermont WIC under a Collaborative Data Transfer and Use Agreement that classifies all shared data as deidentified data about human subjects, with no personally identifiable information per the National Institute of Standards and Technology Special Publication 800-122 and HIPAA (Health Insurance Portability and Accountability Act) deidentification standards. The agreement does not include public access requirements for the data. Deidentified quantitative data (survey responses and aggregated app usage metadata) are stored on HIPAA-compliant servers and in password-protected institutional repositories, and may be made available upon reasonable request to the corresponding author, subject to a data use agreement and approval from the relevant institutional review boards (Vermont Agency of Human Services; Tufts University). Pursuant to the US Department of Agriculture (USDA) cooperative agreement governing this project, the USDA Food and Nutrition Service retains the right to obtain, reproduce, and publish generated data, including in response to Freedom of Information Act requests. Qualitative interview transcripts are not available due to the risk of participant reidentification given the small sample sizes and rural study setting. The Daily Drop app was developed by GameTheory Co as proprietary software; source code is not publicly available.
